# The impact of self-stigma on college students' attitudes toward professional psychological help-seeking: serial-mediated effects of discrimination perceptions and core self-evaluations

**DOI:** 10.3389/fpsyg.2025.1630323

**Published:** 2025-09-10

**Authors:** Daoliang Chen, Yiling Wu, Ling Qian, Ying Zhou

**Affiliations:** ^1^Psychological Health Education Consultation Center, Jinhua University of Vocational Technology, Jinhua, Zhejiang, China; ^2^School of Business, Jinhua University of Vocational Technology, Jinhua, Zhejiang, China

**Keywords:** self-stigma, discrimination perceptions, core self-evaluation, professional psychological help-seeking attitudes, college students

## Abstract

**Purpose:**

To explore the role of self-stigma as predictor of college students' attitudes toward professional psychological help-seeking, as well as the separate and serial mediating roles of discrimination perceptions and core self-evaluations between the two.

**Methods:**

A survey of 574 college students was conducted using the Psychological Help-Seeking Stigma Scale, the Perception of Discrimination Scale, the Core Self-Esteem Scale, and the Attitudes toward Professional Psychological Help-Seeking Scale; descriptive statistics and correlation analyses were conducted using SPSS 25.0; and mediation model tests were conducted using Mplus 8.0.

**Results:**

Self-stigma negatively predicted college students' attitudes toward professional psychological help-seeking (β = −0.13, *p* < 0.01), and discrimination perceptions and core self-evaluations had a significant serial mediation between self-stigma and attitudes toward professional psychological help-seeking, with 95% confidence intervals of [−0.06, −0.01] and mediation effect sizes of −0.04.

**Conclusion:**

Self-stigma can not only directly predict college students' attitudes toward professional psychological help-seeking, but also indirectly predict college students' attitudes toward professional psychological help-seeking through the serial -mediated effects of discrimination perceptions and core self-evaluations.

## Introduction

Professional psychological help-seeking attitudes refer to individuals preferences or inclinations toward resolving psychological or emotional difficulties by seeking assistance from professional counselors or psychologists (Hao and Liang, 2015). Research has shown that these attitudes are a significant predictor of professional help-seeking behaviors (Hao and Liang, 2007a). Individuals with more positive attitudes toward professional psychological services are more likely to engage in such behaviors, which can effectively address their psychological issues and enhance their mental wellbeing (Seyfi et al., 2013). Therefore, exploring the factors influencing college students' professional psychological help-seeking attitudes and understanding the underlying mechanisms are of critical theoretical and practical importance for promoting help-seeking behaviors and improving mental health outcomes in this population.

In recent years, mental health issues among college students have garnered increasing attention from society. To meet students' mental health needs, most universities have established psychological counseling centers staffed with qualified counselors offering free services (Seidel et al., 2020). Some institutions have gone further by collaborating with medical schools or specialized psychiatric hospitals, inviting professional doctors to provide regular, on-campus psychological services. Moreover, many counseling centers have been continuously upgrading their facilities, diversifying the services offered, and expanding their teams of professionals to better address students' individualized needs. However, despite the growing availability of resources, only a small proportion of students seek professional psychological assistance when faced with emotional or psychological challenges (Yong et al., 2023). Emerging research suggests that innovative approaches like digital psychoeducation tools (Chookerd et al., 2025) and AI-driven screening methods (Tuan et al., 2024) may help bridge this utilization gap by making mental health support more engaging and accessible.

This discrepancy highlights that merely increasing the supply of psychological services may not be sufficient to significantly improve students' willingness to seek professional help. The decision to seek professional assistance is not only influenced by the availability and accessibility of services but is also closely tied to students' attitudes toward psychological help-seeking. Students behavior in seeking professional psychological help are influenced by various factors, with one of the most crucial being their attitude toward help-seeking (Hao and Liang, 2015). A positive attitude toward seeking professional help significantly increases the likelihood of students engaging in such behaviors. Recent educational studies highlight how supportive learning environments can mitigate help-seeking barriers (Almarashdi, 2024). However, social stigma and negative judgments of help-seeking behaviors can have a profound impact on students. When confronted with discrimination, students often internalize these negative evaluations as a sense of shame, which not only affects their attitudes toward seeking help but also hinders their actual help-seeking actions (Liang et al., 2020).

Professional psychological help-seeking stigma can be categorized into public stigma and self-stigma (Hao and Liang, 2011). Public stigma refers to the social prejudice and discrimination toward those who seek psychological help, while self-stigma refers to the process in which individuals internalize these negative social perceptions when considering whether to seek help. Specifically, self-stigma occurs when individuals internalize social stigma, forming negative beliefs about seeking psychological assistance (Vogel et al., 2013). The World Health Organization (WHO) states that “social stigma and associated discrimination are the greatest barriers to the recovery of individuals with mental health disorders” (Yang et al., 2007). In response to these stigmas, individuals often adopt coping strategies such as self-concealment, self-devaluation, and avoidance of social interaction. However, these strategies may be detrimental to the development of a positive self-concept and trigger negative emotional experiences (Kroska and Harkness, 2006).

In studies on self-stigma among individuals with mental health conditions, Li et al. (2010) conducted basic research that examined the negative effects of self-stigma. The results showed that all individuals with mental health disorders experienced varying degrees of self-stigma. Meanwhile, this internalized stigma manifested as negative attitudes toward life, denial of personal worth, social avoidance, and the acceptance of stereotypes, which in turn led to decreased self-efficacy, lowered self-esteem, increased depressive symptoms, social withdrawal, and a reluctance to seek help (Li and Gao, 2009).

Self-stigma is a crucial factor affecting the attitudes of university students toward professional psychological help-seeking (Jennings et al., 2015). Moreover, self-stigma may also indirectly affect these attitudes through other mediating factors. According to the Modified Labeling Theory (Hunter et al., 2017), when individuals are categorized into a specific social group, they may face expectations from socio-cultural perceptions. When an individual chooses to seek help for psychological issues, they risk being viewed as engaging in deviant behavior. Many parents and educators, due to a lack of mental health knowledge, may perceive seeking psychological help as deviating from societal norms, and as a result, they may label the individual negatively. Such labeling is often associated with self-devaluation and external discrimination (Chung, 2004).

Discrimination perception refers to the feeling of being unfairly or negatively treated by others because of own identity or the group to which they belong (Major et al., 2002). This can manifest in discriminatory behaviors, unequal social systems, or negative societal attitudes toward specific groups. Despite the increasing awareness and understanding of mental health issues, and a growing tolerance for psychological problems, the inherent biases and stigmatization of mental illnesses have not been fully eradicated (Lo et al., 2021). According to the Modified Labeling Theory, individuals who internalize stigmatizing beliefs and view themselves as worthless due to the labels attached to their mental health issues often exhibit self-devaluing tendencies. These individuals are more likely to experience increased external discrimination, which ultimately intensifies their discrimination perception (Link et al., 1989).

The psychological effect of self-stigma, often referred to as self-shame, significantly disrupts individuals' life goals and lowers their quality of life, thereby hindering their willingness to seek help (Jiang and Xia, 2006). In this context, individuals may avoid professional psychological help-seeking due to the fear of being labeled negatively. Therefore, discrimination perception may play a mediating role in the relationship between self-shame and attitudes toward professional psychological help-seeking. Specifically, individuals' self-devaluation and heightened perception of social discrimination may amplify their internalized sense of shame, which further diminishes their positive attitude toward professional psychological help-seeking (Alluhaibi and Awadalla, 2022). This mechanism indicates that discrimination perception not only directly impacts an individual's psychological state but also indirectly influences their willingness to seek help by altering their social identity and sense of self-worth (Ziersch et al., 2020). Core self-evaluation is an individual's fundamental assessment of their own value and abilities, encompassing four key personality traits: self-esteem, neuroticism, locus of control, and general self-efficacy (Judge et al., 2002). Research has shown that seeking help may lead to feelings of self-threat, as individuals may perceive their need for assistance as a sign of weakness (Mao and Sun, 2011). Individuals with higher core self-evaluations, who possess a more positive view of their abilities and worth, are generally more confident in handling interpersonal situations and in seeking help from others, thus experiencing less perceived self-threat (Sun and Mao, 2013). Studies suggest that individuals with high core self-evaluations tend to engage in more proactive behaviors compared to those with lower core self-evaluations (Grant and Ashford, 2008).

Symbolic interaction theory posits that individuals often rely on evaluations of others toshape their self-concept (Brownfield and Thompson, 2005). When individuals perceive negative feedback or evaluations from others, their self-assessment tends to decrease, which, in turn, affects their perception of their own abilities and worth (Jhang, 2017). This phenomenon can lead to a diminished sense of self-esteem and an increased sense of self-shame. Therefore, it can be predicted that core self-evaluation may play a mediating role in the relationship between self-shame and attitudes toward professional psychological help-seeking. In this context, individuals with lower core self-evaluations are more likely to internalize negative feedback (Faith and Shirley, 2018) and view seeking help as a threat to their self-concept, which hinders their willingness to seek professional assistance.

Core self-evaluations, therefore, not only directly influence an individual's confidence in their own abilities but also interact with self-stigma to shape their attitudes toward help-seeking behaviors. Individuals with high core self-evaluations are better equipped to cope with perceived social stigma, reducing the impact of self-shame on their willingness to seek psychological help. In contrast, individuals with low core self-evaluations may be more susceptible to negative societal feedback, leading to increased self-stigma and a more negative attitude toward seeking professional support (Xinran, 2023).

**Figure 1:**
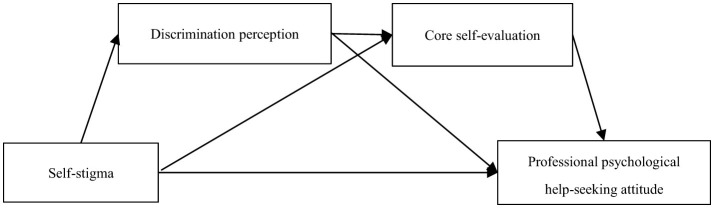
Serial mediation hypothesis model path.

Based on the above discussion, the present study aims to examine the predictive roles of self-stigma, discrimination perception, and core self-evaluation in shaping college students' attitudes toward professional psychological help-seeking. Additionally, this study also explored the mediating effects of discrimination perception and core self-evaluation in these relationships, focusing on a sample of college students in China. Given that gender may influence attitudes toward seeking professional psychological help (Mackenzie et al., 2006), it was included as a control variable in the model. Based on theoretical frameworks and empirical findings from previous research, the following hypothesized model was proposed (see Figure 1).

## Methods

### Participants

Using a convenience sampling method, a total of 574 college students from universities in Zhejiang Province, China, were selected as participants. Demographic composition is shown in Table 1.

**Table 1:** Demographic characteristics of participants (*N* = 574).

**Demographic variable**	**Category**	***n***	**Percentage (%)**
Gender	Male	156	27.18
	Female	418	72.82
Academic year	Freshman	176	30.66
	Sophomore	292	50.87
	Junior	106	18.47
Residential origin	Rural	436	75.95
	Urban	138	24.04
Academic major	Liberal arts	125	21.78
	Science	213	37.11
	Arts and physical education	236	41.11
Only child status	Yes	201	35.02
	No	373	64.98
Family economic status	Affluent	6	1.05
	Well-off	78	13.59
	Average	399	69.51
	Impoverished	91	15.85

### Measurement tools

#### Psychological help-seeking stigma scale

The Psychological Help-Seeking Stigma Scale (Vogel et al., 2013), revised by Hao and Liang for Chinese college students (Hao and Liang, 2011), was used to measure individuals' sense of stigma for seeking professional psychological help. The scale consists of 10 items, including two dimensions of self-stigma and public stigma. Each item is rated on a 5-point Likert scale, ranging from 1 (“completely disagree”) to 5 (“completely agree”). For example, if I seek professional psychological help, I will feel imperfect. In the present study, the self-stigma dimension demonstrated high internal consistency, with a Cronbach's alpha coefficient of 0.90.

#### Discrimination perception scale

The Perception of Discrimination Questionnaire (Shen et al., 2009), developed by Shen Jiliang et al. and validated in Chinese adolescent samples, was utilized in this study. The questionnaire comprises six items across two dimensions: individual discrimination intuition and group discrimination perception. Responses are measured on a 5-point Likert scale, ranging from 1 (“not at all”) to 5 (“completely”). For example, I feel like I'm being treated unfairly. In this study, the scale demonstrated excellent internal consistency, with a Cronbach's alpha coefficient of 0.91.

#### Core self-evaluation scale

The Core Self-Evaluation revised scale by Du et al. (2012), which was adapted for Chinese populations, was used in this study. It consists of 10 items rated on a 5-point Likert scale, with responses ranging from 1 (“completely disagree”) to 5 (“completely agree”). Items 2, 3, 5, 7, 8, and 10 are reverse-scored, and higher total scores indicate higher levels of individual core self-evaluations. A sample item is “I believe I can succeed in life.” In the present study, the scale demonstrated good internal consistency, with a Cronbach's alpha coefficient of 0.80.

#### Professional psychological help-seeking attitudes scale

This study used a revised Professional Psychological Help-Seeking Attitude Scale by Hao and Liang (2007b), validated in Chinese college students. The revised scale was divided into four dimensions: self-perception, confidence in mental health professionals, interpersonal openness, and tolerance of stigma. The scale is also scored on a 5-point Likert, with 1 indicating “completely disagree” and 5 indicating “completely agree.” For example, although there are many clinics for people with mental health issues, I don't really trust them. Eighteen of the questions are reverse scores, and the total score of all the options will be added up to the total score. The higher the score, the more positive the attitude toward professional psychological help. In this study, the Cronbach's alpha coefficient of this scale was 0.77.

### Research process and data processing

All questionnaires were administered collectively after obtaining the informed consent of the participating university students. The testing process was conducted class by class, with each session overseen by a trained teacher who served as the primary examiner. These teachers had undergone standardized training to ensure consistency and reliability in data collection. Once the questionnaires were collected and the data organized, descriptive statistics and correlation analyses were performed using SPSS 25.0. To examine the mediation models, advanced statistical analyses were conducted in Mplus 8.0, ensuring a robust and comprehensive evaluation of the proposed relationships among the variables.

## Results

### Common method deviation test

The Harman one-way test was employed to assess common method bias across all items from the four scales: self-stigma, discrimination perception, core self-evaluation, and attitudes toward professional psychological help-seeking. The analysis identified eight factors with eigenvalues greater than 1, with the largest factor accounting for 22.38% of the variance. Since this value is well below the threshold of 40%, it can be concluded that the scales used in this study are not significantly affected by common method bias.

### Descriptive statistics and correlational analysis

The results revealed several significant relationships among the study variables. Self-stigma demonstrated a significant positive correlation with discrimination perceptions (indicating that higher self-stigma is associated with stronger perceptions of discrimination) and significant negative correlations with both core self-evaluations and attitudes toward professional psychological help-seeking (suggesting that higher self-stigma is linked to lower self-assessments and less favorable help-seeking attitudes). Similarly, discrimination perceptions were significantly negatively correlated with both core self-evaluations and attitudes toward professional psychological help-seeking, indicating that higher perceptions of discrimination are associated with reduced self-evaluations and more negative attitudes toward seeking help. Furthermore, core self-evaluations were found to be significantly positively correlated with attitudes toward professional psychological help-seeking, highlighting that individuals with higher self-evaluations tend to hold more favorable attitudes toward seeking professional help. Detailed results are presented in Table 2.

**Table 2:** Descriptive statistics and correlation analysis results.

**Variable**	***M ±SD***	**1**	**2**	**3**	**4**	**5**
1 Self-stigma	2.37 ± 0.83	1				
2 Discrimination perception	2.48 ± 0.79	0.48^***^	1			
3 Core self-evaluation	3.25 ± 0.56	−0.50^***^	−0.59^***^	1		
4 Professional psychological help-seeking attitude	3.22 ± 0.34	−0.35^***^	−0.47^***^	0.41^***^	1	
5 Gender		0.02	−0.06	−0.01	0.15^***^	1

*** p < 0.001,

^**^p < 0.01.

### Mediation model testing

In this study, gender was found to be significantly associated with attitudes toward professional psychological help-seeking, consistent with previous research findings that have also demonstrated this relationship (Hao and Liang, 2007a). Consequently, gender was included as a control variable in the mediation model.

**Figure 2:**
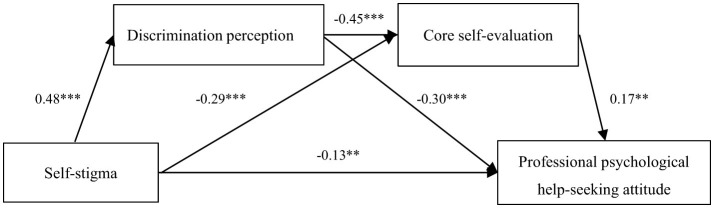
Serial mediation model of self-stigma, discrimination perception, core self-evaluation, and professional psychological help-seeking attitude. ***p < 0.001, **p < 0.01.

First, self-stigma was a significant negative predictor of professional psychological help-seeking attitudes (β = −0.13, *p* < 0.01). Second, mediation effects analyses with self-stigma as a predictor variable, attitudes toward professional psychological help-seeking as an outcome variable, and discrimination perceptions and core self-evaluations as mediator variables showed a good model fit [χ^2^/df = 1.92, CFI = 1.00, TLI = 0.99, RMSEA (90% CI) = 0.04 (0.00–0.10), SRMR = 0.02]. The results of the path analyses indicated that self-stigma significantly and positively predicted discrimination perceptions (β = 0.48, *p* < 0.001), discrimination perceptions significantly and negatively predicted core self-evaluations (β = −0.45, *p* < 0.001) and attitudes toward professional psychological help-seeking (β = −0.30, *p* < 0.001), and that core self-evaluations significantly and positively predicted attitudes toward professional psychological help-seeking (β = 0.17, *p* < 0.01; see Figure 2).

Confidence intervals for the mediating effect sizes were estimated using the bias-corrected non-parametric percentile Bootstrap method with 1,000 repetitive samples, and it was found that the mediating effect value of discrimination perceptions between self-stigma and attitudes toward professional psychological help-seeking was −0.14, with 95% confidence intervals of (−0.19, −0.09), and that the mediating effect of discrimination perceptions was significant. The mediation effect value of core self-evaluation between self-stigma and attitudes toward professional psychological help-seeking was −0.05 with a 95% confidence interval of (−0.09, −0.01), and the mediation effect of core self-evaluation was significant. In addition, the serial mediation effect between discrimination perception and core self-evaluation was −0.04, with a 95% confidence interval of (−0.06, −0.01), suggesting that self-stigma can influence individuals' attitudes toward professional psychological help-seeking through influencing discrimination perception and then core self-evaluation (see Table 3).

**Table 3:** Mediation effect test.

**Path**	**Effect value**	**95% confidence interval of path effect value**
Self-stigma-discrimination perception- Professional psychological help-seeking attitude	−0.14	−0.19, −0.09
Self-stigma-core self-evaluation-professional psychological help-seeking attitude	−0.05	−0.09, −0.01
Self-stigma-Discrimination perception- Core self-evaluation-Professional psychological help-seeking attitude	−0.04	−0.06, −0.01

## Discussion

The results of this study showed that college students' self-stigma was a significant negative predictor of attitudes toward professional psychological help-seeking, a result consistent with previous research (Hao, 2009; Tang and Wen, 2015) Corrigan et al. suggest that self-stigma manifests itself in a three-level model: stereotype congruence, self-identification, and decreased self-esteem (Corrigan, 2002). The higher the level of self-stigma, the more likely college students were to lead to low self-esteem and avoidant behavior. They feel that if they seek professional psychological help, it will trigger negative evaluations and they will feel discriminated against by society. Even if they know that seeking professional psychological help is an effective way to solve their psychological problems, they still choose to avoid it in case to becoming a target of discrimination. It is recommended that colleges and universities should focus on reducing stigma in mental health education and improve students acceptance of psychological help through publicity and education activities. At the same time, we suggest strengthening privacy protection mechanisms in mental health services to reduce students' perception of discrimination and improve service utilization.

Discrimination perceptions mediate significantly between self-stigma and professional psychological help-seeking attitudes among college students. Self-stigma affects professional help-seeking attitudes through discrimination perceptions. Despite the increasing popularity of mental health knowledge and the increasing understanding and tolerance of psychological problems and mental illnesses, the inherent prejudice against “psychological help-seeking” or “mental illnesses” has not been completely eradicated. Viewing oneself as stigmatized and worthless due to an illness, as perceived through the lens of others, can foster self-deprecation and amplify feelings of being discriminated against, ultimately heightening perceptions of discrimination (Mittal et al., 2012). Learned helplessness theory suggests that perceptions of discrimination can lead individuals to feel powerless and unable to alter negative external evaluations or influences, fostering a profound sense of helplessness or loss that ultimately hinders help-seeking behaviors (Ruggiero and Taylor, 1997).

Similarly, core self-evaluation mediated significantly between self-stigma and professional psychological help-seeking attitudes among college students. Self-stigma affects professional psychological help-seeking attitudes through core self-evaluations. Individuals need to draw on the feedback evaluations of others to build their self-concept, and self-stigma tends to internalize stigmatized views held by the public; therefore, asking for help from others can bring about feelings of self-threat (Mao and Sun, 2011). Individuals with different levels of core self will behave differently in the face of threatening perceptions, with those with high core self-evaluations being more self-confident and able to resist the threatening perceptions associated with help-seeking, and may show a tendency to engage in more proactive behaviors (Jhang, 2017). Therefore, in practical work, more psychological support and emotional guidance can be provided to students with low core self-evaluation, thereby enhancing their confidence and willingness to seek help. In addition to the significant mediating role alone, the present study found that discrimination perceptions and core self-evaluations serially mediated the relationship between college students' self-stigma and their attitudes toward professional psychological help-seeking. This result validates the idea of modified labeling theory that when individuals perceive that they are psychologically disturbed and want to seek professional psychological help, they tend to expect that they will be devalued and discriminated against. In order to avoid being ostracized, they adopt various coping strategies which are detrimental to the development of self-concept and may lead to a decrease in the individual's perceived social support (Fan et al., 2012), a decrease in self-efficacy, a decrease in core self-appraisal (Xie et al., 2016), and the development of negative emotional experiences. Thus, under the influence of anticipated devaluation and discrimination, individuals are triggered to have high perceptions of discrimination and low core self-evaluations, which ultimately affect their attitudes toward seeking professional psychological help.

In summary, this study clarifies the role of discrimination perception and core self-evaluation as mediator in the relationship between self-stigma and attitudes toward professional psychological help-seeking. It also provides a theoretical basis for developing interventions to improve college students' attitudes toward professional psychological help-seeking. However, this study has several limitations that warrant further discussion. First, the mechanism through which self-stigma affects attitudes toward professional psychological help-seeking is complex, and focusing solely on discrimination perception and core self-evaluation may not fully capture the intrinsic connection. Future research should consider additional variables, such as social support, cultural influences, or personality traits, to provide a more comprehensive understanding. Secondly, this research adopts a cross-sectional study, which means that the causality between the variables is uncertain, so the follow-up should be supplemented with additional longitudinal data to conduct the study. Moreover, the study sample had its own constraints. For instance, it was limited to a specific geographic region, which may restrict the generalizability of the findings. The demographic data collected were also limited, potentially omitting important moderating factors like socioeconomic status, race. Finally, the reliance on self-reported measures could introduce bias, such as social desirability effects. Finally, due to the limitation of research resources and sample size, variables such as social support, mental health knowledge and previous counseling experience were not included in this study. Addressing these issues in future research will help enhance the validity and applicability of the findings.

## Conclusion

This study identified self-stigma as a significant negative predictor of attitudes toward professional psychological help-seeking. Furthermore, discrimination perceptions and core self-evaluations were found to function as serial mediators in the relationship between self-stigma and attitudes toward professional psychological help-seeking.

By conducting an in-depth analysis, the study not only reaffirmed the negative impact of self-stigma on college students' attitudes toward seeking professional psychological help but also uncovered the underlying mechanisms driving this effect. These findings provide some new ideas for the intervention path to improve college students' attitudes toward professional psychological help-seeking. Specifically, professional psychological help-seeking attitudes can be effectively enhanced by reducing discriminatory cognition and strengthening core self-evaluation. For example, colleges and universities can combine anti-stigma publicity with core self-evaluation training, reduce students' discrimination perceptions of psychological help-seeking through case education, and improve their core self-evaluations through group counseling, thereby improving their help-seeking attitudes. Targeted intervention on these key factors may cultivate a more positive help-seeking attitude and ultimately promote the mental health of college students.

## Data Availability

The raw data supporting the conclusions of this article will be made available by the authors, without undue reservation.
